# Sleep disturbances in ADHD: investigating the contribution of polygenic liability for ADHD and sleep-related phenotypes

**DOI:** 10.1007/s00787-021-01931-2

**Published:** 2022-01-07

**Authors:** Katie J. S. Lewis, Joanna Martin, Alice M. Gregory, Richard Anney, Anita Thapar, Kate Langley

**Affiliations:** 1 MRC Centre for Neuropsychiatric Genetics and Genomics, Cardiff University, Cardiff, UK; 2 Department of Psychology, Goldsmiths, University of London, London, UK; 3 School of Psychology, Cardiff University, Cardiff, UK

**Keywords:** Sleep, ADHD, Comorbidity, Polygenic scores

## Abstract

Sleep disturbances are common in attention deficit hyperactivity disorder (ADHD) and associated with poor outcomes. We tested whether, in children with ADHD, (1) polygenic liability for sleep phenotypes is over- or under-transmitted from parents, (2) this liability is linked to comorbid sleep disturbances, and (3) ADHD genetic risk is associated with comorbid sleep disturbances. We derived polygenic scores (PGS) for insomnia, chronotype, sleep duration, and ADHD, in 758 children (5–18 years old) diagnosed with ADHD and their parents. We conducted polygenic transmission disequilibrium tests for each sleep PGS in complete parent–offspring ADHD trios (*N* = 328) and an independent replication sample of ADHD trios (*N* = 844). Next, we tested whether insomnia, sleep duration, and ADHD PGS were associated with co-occurring sleep phenotypes (hypersomnia, insomnia, restless sleep, poor sleep quality, and nightmares) in children with ADHD. Children’s insomnia and chronotype PGS did not differ from mid-parent average PGS but long sleep duration PGS were significantly over-transmitted to children with ADHD. This was supported by a combined analysis using the replication sample. Insomnia, sleep duration, and ADHD PGS were not associated with comorbid sleep disturbances. There is weak evidence that children with ADHD over-inherit polygenic liability for longer sleep duration and do not differentially inherit polygenic liability for insomnia or chronotype. There was insufficient evidence that childhood sleep disturbances were driven by polygenic liability for ADHD or sleep traits, suggesting that sleep disturbances in ADHD may be aetiologically different to general population sleep phenotypes and do not index greater ADHD genetic risk burden.

**Supplementary Information:**

The online version contains supplementary material available at 10.1007/s00787-021-01931-2.

## Introduction

Attention deficit hyperactivity disorder (ADHD) is a common and impairing neurodevelopmental condition, which frequently co-occurs with sleep disturbances. Children with ADHD are more likely to experience sleep disorder symptoms such as insomnia, hypersomnia, and delayed sleep phase syndrome compared to children without ADHD [1]. These sleep disturbances are important to identify and treat, as they are associated with ADHD persistence [2], reduced quality of life [3], and reduced individual and family functioning [4]. Furthermore, ADHD is associated with other aspects of sleep and sleep duration may moderate treatment response [5]. There is also evidence that improving sleep could improve ADHD symptoms [6, 7]. It is, therefore, important to determine why ADHD and sleep disturbances co-occur and identify which individuals with ADHD are at heightened risk for developing such problems.

Sleep disturbances may arise in individuals with ADHD due to shared genetic influences. This theory has been supported by twin studies [2] and in a genome-wide association study (GWAS) of ADHD, which identified a positive genetic correlation between ADHD and insomnia [8]. Polygenic scores are an individual index of genetic liability for a phenotype of interest and can be used to better understand the genetic relationship between ADHD and sleep problems. For example, recent studies have found that ADHD symptoms in children in the general population are associated with polygenic scores for insomnia [9] and narcolepsy [10]. Additionally, ADHD polygenic scores are associated with sleep disturbances in children in the general population, particularly excessive somnolence and difficulties initiating and maintaining sleep [11]. Further investigation is needed of whether polygenic liability for several different sleep phenotypes is associated with ADHD clinical diagnosis in children. Case–control studies can be confounded by population stratification and other factors (e.g., differences between cases and controls in age, sex, medical history, educational attainment or socioeconomic background). An alternative method which is robust to these factors is the polygenic transmission disequilibrium test (pTDT) [12]. The pTDT can be used to test whether common variant liability for complex traits is over- or under-transmitted from parents to children ascertained on the basis of a particular disorder. Such differential transmission of polygenic liability related to sleep phenotypes would further support shared genetic liability between ADHD and sleep phenotypes.

A further question is whether the common polygenic liability to sleep traits in general populations also indexes comorbid sleep disturbances in children with ADHD. This is important to test because it is possible that these sleep disturbances arise from different mechanisms to those in the general population. For example, they could arise due to ADHD symptoms (e.g. a racing mind), which are indexed by genetic liability to ADHD, rather than a genetic liability to sleep phenotypes. There are also environmental factors associated with ADHD that increase the likelihood of experiencing sleep disturbances (e.g. socioeconomic status, medication) [13].

Finally, a related question concerns why children with ADHD are at heightened risk for developing sleep problems. As sleep problems in children with ADHD are associated with symptom severity, co-occurring disorders and neurocognitive deficits [14, 15], these problems may index neurodevelopmental or clinical severity. Previous work has shown that genetic risk for ADHD is associated with other comorbid difficulties in children with ADHD (e.g. conduct disorder) [16]. In addition, sleep disturbances are common in the context of many other psychiatric disorders such as depression [17], which also share polygenic liability with sleep problems [17, 18] and ADHD [19, 20]. Polygenic scores for ADHD and psychiatric disorders can be used to test whether sleep disturbances observed in children with ADHD are an indicator of neurodevelopmental or clinical severity.

Our overall aim in this study was to test whether sleep disturbances in children with ADHD are driven by polygenic liability for general population sleep phenotypes and are an indicator of ADHD severity. First, we hypothesised that polygenic liability for insomnia would be over-transmitted from parents to children diagnosed with ADHD, and that polygenic liability for sleep duration and chronotype (i.e. delayed sleep phase) would be over- or under-transmitted. These expectations were based on previously estimated genetic correlations between ADHD and sleep phenotypes (positive genetic correlation with insomnia and no evidence of correlation in either direction for other sleep phenotypes) in primarily case–control samples [8]. Second, we hypothesised that greater polygenic liability for sleep phenotypes (measured in adult populations) would be associated with increased risk of insomnia, poor sleep quality and hypersomnia in children with ADHD. Finally, we hypothesised that a greater polygenic risk for ADHD would increase the risk of sleep disturbances in children with ADHD. In exploratory analyses, we examined whether greater genetic risks for other disorders commonly comorbid with ADHD (i.e. anxiety, depression, and Autism Spectrum Disorder (ASD)) are associated with increased risk of sleep disturbances in children with ADHD.

## Method

The sample comprised children with ADHD (aged 5–18 years), recruited from out-patient clinics (including Child and Adolescent Mental Health Services and Paediatrics) in Wales. Parents and children aged 16 years or older provided written informed consent and children under 16 years old provided assent to take part in the study. Ethical approval was obtained from the North West England and Wales Multicentre Research Ethics Committees and the Cardiff University School of Medicine Research Ethics Committee.

ADHD diagnosis was confirmed using the Child and Adolescent Psychiatric Assessment (CAPA; [21]), a semi-structured diagnostic interview completed with parents by trained and clinically supervised psychologists. Children in the sample who were aged ≥ 12 years were also interviewed using the CAPA. Presence of the 18 DSM-IV/ICD-10 ADHD symptoms, 2 additional ADHD symptoms for DSM-III-R, impairment, and age of onset of symptoms were ascertained. Symptom pervasiveness was confirmed via teacher report using the Child ADHD Teacher Telephone Interview (CHATTI) [22] or Conners’ Teacher Rating Scale [23]. Children were included if they met ADHD diagnostic criteria according to (the then current) DSM-IV at the time of the interview, with a small subset (*N* = 72) from an earlier part of the study who met DSM-III-R criteria (where teacher information on symptom pervasiveness was not available).

Presence of sleep disturbances was also assessed using the CAPA criteria. The following items were assessed: any insomnia of ≥ 1 h of being unable to get to sleep (including any of the following: delayed sleep onset, night waking, early morning waking), hypersomnia, restless sleep, poor sleep quality (defined in the CAPA as being inadequately rested by sleep), and nightmares. All sleep disturbance items were coded as binary variables (0 = absent, 1 = present). For children aged > 11 years who also completed the CAPA, a symptom was considered present if either the parent or child reported it. The presence of nightmares was only assessed in the parent CAPA. Parents also reported whether their children were currently taking any medication for ADHD (i.e. stimulants or risperidone) or for sleep disturbances (e.g. melatonin). Children with missing parent CAPA information for sleep variables (from a part of the study when this section was not included) were excluded (*N* = 101) from that part of the analysis.

Children and both biological parents were asked to provide a DNA sample (saliva or blood). The samples were genotyped in several batches, with rigorous quality control (QC) procedures (see Supplement). We used publicly available large discovery GWAS summary statistics based on studies of individuals of European ancestries that were independent of our sample, to derive polygenic scores (PGS) using PLINK version 1.9 [24]. We used the following primary discovery phenotypes: frequent insomnia symptoms (129,270 cases and 108,357 controls) [18], chronotype (252,287 cases and 150,908 controls) [25], self-reported sleep duration (total length of hours: 446,118 individuals; short sleep duration: 106,192 cases and 305,742 controls; long sleep duration: 34,184 cases and 305,742 controls) [26], and ADHD (18,378 cases and 29,113 controls; after excluding our sample) [8]. For the exploratory analyses, we used the following discovery phenotypes: anxiety disorders (31,977 cases and 82,114 controls) [27], major depressive disorder (MDD; 59,851 cases and 113,154 controls) [28], and autism spectrum disorder (ASD; 18,382 cases and 27,969 controls) [29].

For each of these GWAS, common variants (Minor allele frequency (MAF) > 5%) that overlapped with our sample were used to calculate PGS for each individual by summing the number of alleles (weighted by the log of the odds ratio) across the set of single nucelotide polymorphisms (SNPs) in PLINK (using command –score). We then used the PGS-PCA approach [30], in which a principal component analysis was conducted on scores derived using 7 *p* value thresholds and the first principal component was extracted and standardised using z-score transformations (further details in Supplement).

### Analyses

For aim 1, we used the pTDT [12], in which we compared the mean proband PGS to the mean parental mid-point PGS. This tests for over- or under-transmission of alleles for a given phenotype in the proband compared to what would be expected given the average of the parental genotypes. For aim 2, we tested whether insomnia PGS were associated with insomnia and poor sleep quality and whether sleep duration PGS were associated with insomnia, poor sleep quality and hypersomnia. For aim 3, we tested whether ADHD PGS were associated with any of the sleep disturbances. We also performed exploratory analyses testing whether PGS for anxiety, depression, or ASD were associated with any sleep disturbances.

The sample included 40 families with full- or half-siblings. Only one child per family was selected for pTDT analyses. For all other analyses, we accounted for genetically related samples using a sandwich estimator to correct the standard errors of regression coefficients, using generalised estimating equations implemented in the *drgee* R package. For all PGS analyses, the top 5 PCs, genotyping batch and child’s age at assessment were included as covariates. Nagelkerke *R*^2^ differences between null and full models were calculated to obtain estimates of variance explained, and we applied correction for multiple testing using a false discovery rate (FDR) threshold of 0.05. We ran several sensitivity analyses: (a) using only CAPA information from parents, (b) including sex and ADHD medication as additional covariates, and (c) excluding children taking any medication for sleep.

### Replication sample

Replication data for Aim 1 were available from the International Multicentre ADHD Genetics (IMAGE) study, consisting of 844 complete trios of ADHD probands and both biological parents [31] (see Supplement for details of sample QC). PGS were calculated using GWAS of sleep phenotypes, using the same method as described above. Secondary phenotype data relating to sleep phenotypes were limited in this sample and were not analysed.

ADHD diagnosis was confirmed in IMAGE using the ADHD section of the Parental Account of Childhood Symptoms [32], a semi-structured interview in which parents rate the frequency or severity of their child’s ADHD symptoms (details in Supplement). Further information on study procedures is outlined elsewhere [33].

To increase the power of the pTDT analysis, we also performed a joint analysis of the primary and replication samples to give an overall estimate of the effect sizes across both samples. To do this, we combined the deviation of children’s PGS from their parents’ average PGS across the two datasets and performed the pTDT in the combined sample (*N* = 1172 trios).

## Results

758 children with diagnosed ADHD had available information about sleep disturbances and genetic data after QC and were included in the analyses. Table 1 displays the frequencies of sleep problems in the sample, and other descriptive variables. Sleep disturbances were common, with restless sleep being the most commonly reported problem.

**Table 1:** Primary ADHD sample description

Binary phenotypes	*N* (%)
Sex (female)	101(13.3)
Any insomnia	192(25.6)
Restless sleep	294(55.9)
Poor sleep quality	104(19.7)
Nightmares	97(18.4)
Hypersomnia	31(4.1)
Low family social status	315(48.5)
Low family income	269(62.9)
Low parental education	129(28.2)
ADHD medication use	214(30.3)
Sleep medication use	76(14.8)

Continuous phenotypes	Mean(SE)
Child’s age at assessment	10.3(0.1)
Inattention symptoms	7.34(0.062)
Hyperactive-impulsive symptoms	7.72(0.055)
Total ADHD symptoms	15.1(0.09)

**Fig. 1:**
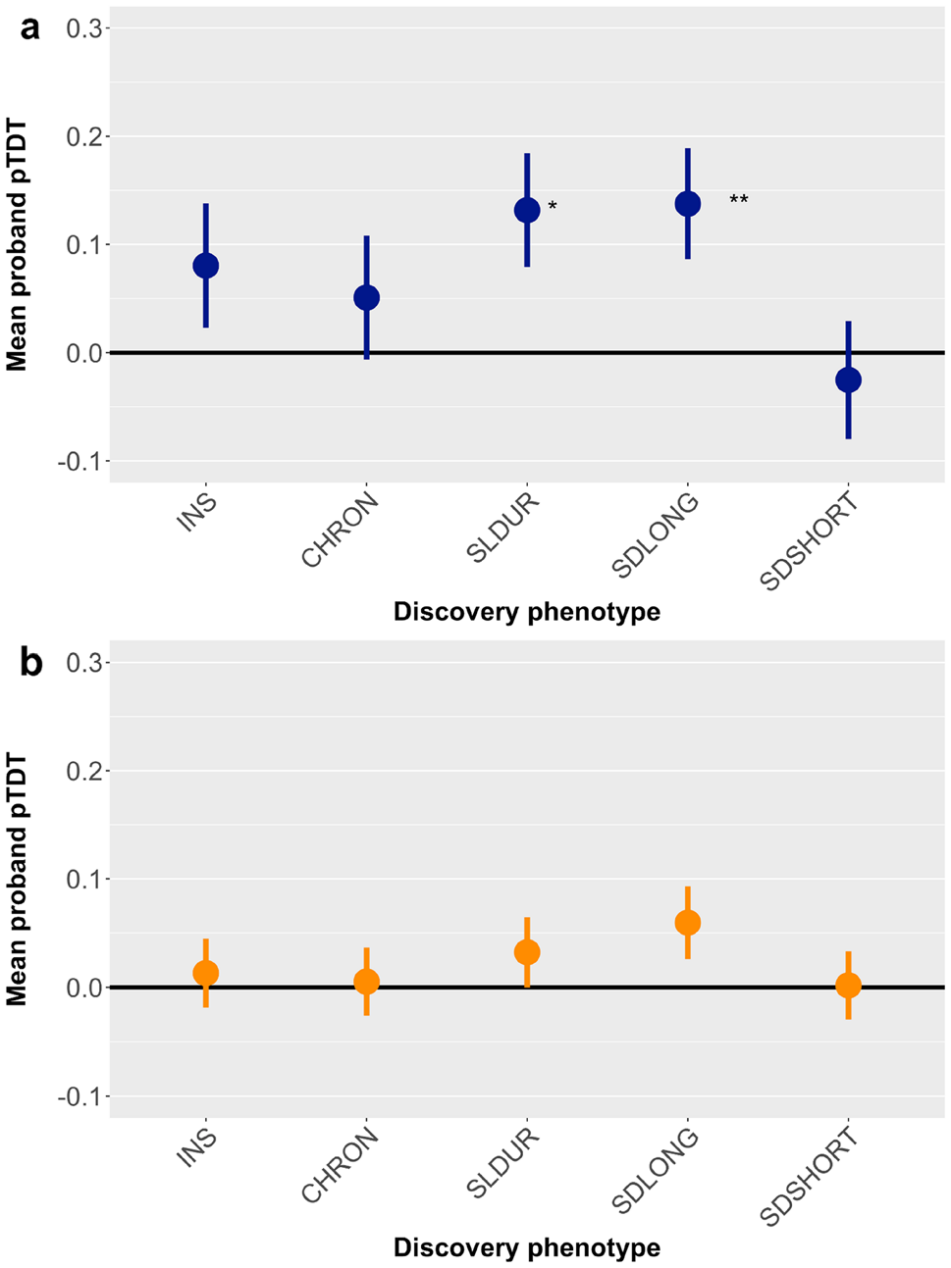
Results of the pTDT analysis displaying the mean deviation of the child’s polygenic score from the mid-parent polygenic score, along with standard errors, in a) the primary sample and b) the replication sample. *INS* insomnia, *CHRON* chronotype, *SLDUR* sleep duration, *SDLONG* sleep duration (long), *SDSHORT* sleep duration (short). *p < 0.05; **p < 0.01

The pTDT analysis of 328 complete parent–offspring trios indicated that polygenic liability for sleep duration was over-inherited by children with ADHD, compared to the average of their parents’ polygenic profiles [mean = 0.13(0.05), p_FDR_ = 0.032]. This association originated specifically from polygenic liability for longer sleep duration [mean = 0.14(0.05), p_FDR_ = 0.032]. There was no evidence of differential transmission of polygenic liability for short sleep duration, insomnia, or chronotype. See Fig. 1a and Table S1 for detailed results.

We used a second sample of 844 children with ADHD from the IMAGE dataset [31], to try to replicate these analyses; see Table S2 for a description of the sample. The results showed a weaker and non-significant result for over-transmission of long sleep duration polygenic liability [mean = 0.06(0.03), *p* = 0.077] (Fig. 1b and Table S1). This result was significant in the combined sample analysis of 1,172 trios [mean = 0.08(0.03), *p* = 0.0039]; see Table S1.

Insomnia PGS were not associated with insomnia in children with ADHD (Table 2). Despite over-transmission of sleep duration polygenic liability to children with ADHD, based on the pTDT analysis, this liability was not associated with co-occurring insomnia, sleep quality, or hypersomnia in the sample (Table 2). ADHD PGS were also not associated with any of the sleep disturbances in the sample (Table 3). Finally, PGS for other commonly comorbid psychiatric disorders (anxiety, depression, and ASD) were also not associated with co-occurring insomnia or other sleep problems (Table 4). The pattern of results did not differ in sensitivity analyses (Tables S3–S5).

**Table 2:** Association of insomnia and sleep duration polygenic scores with sleep disturbances in children with ADHD

Polygenic score	Outcome	*N*	OR(95% CI)	*p*	p_FDR_	*R*^2^
Insomnia	Insomnia	750	1.05(0.88–1.24)	0.61	0.76	0.00055
	Sleep quality	527	1.17(0.94–1.46)	0.16	0.76	0.0056
Sleep duration	Insomnia	750	0.93(0.79–1.10)	0.43	0.76	0.0012
	Sleep quality	527	1.04(0.83–1.29)	0.74	0.81	0.0003
	Hypersomnia	755	1.01(0.69–1.48)	0.96	0.96	0.000013
Sleep duration (long)	Insomnia	750	1.19(1.00–1.40)	0.045	0.50	0.0077
	Sleep quality	527	1.06(0.84–1.33)	0.62	0.76	0.00075
	Hypersomnia	755	0.86(0.61–1.20)	0.36	0.76	0.0032
Sleep duration (short)	Insomnia	750	1.09(0.92–1.29)	0.31	0.76	0.002
	Sleep quality	527	1.12(0.91–1.39)	0.27	0.76	0.0031
	Hypersomnia	755	0.89(0.64–1.23)	0.49	0.76	0.0017

*FDR* false discovery rate, *R*^2^ variance explained by PGS (polygenic score)

**Table 3:** Association of ADHD polygenic scores with sleep disturbances in children with ADHD

Polygenic score	Outcome	*N*	OR(95% CI)		*p*	p_FDR_	*R*^2^
ADHD	Insomnia	750		1.02(0.86–1.20)	0.68	0.95	0.00033
	Restless sleep	526		0.92(0.77–1.09)	0.33	0.95	0.0024
	Sleep quality	527		0.93(0.75–1.15)	0.57	0.95	0.00096
	Nightmares	527		0.95(0.76–1.19)	0.93	0.95	0.000025
	Hypersomnia	755		1.01(0.69–1.50)	0.71	0.95	0.00067

*FDR* false discovery rate, *R*^2^ variance explained by PGS (polygenic score)

**Table 4:** Association of neurodevelopmental/psychiatric polygenic scores with sleep disturbances in children with ADHD

Polygenic score	Outcome	*N*	OR(95% CI)	*p*	p_FDR_	*R*^2^
Anxiety	Insomnia	748	0.97(0.82–1.16)	0.77	0.90	0.00018
	Restless sleep	524	1.05(0.87–1.25)	0.63	0.90	0.0006
	Sleep quality	525	1.04(0.84–1.29)	0.73	0.90	0.00031
	Nightmares	525	1.19(0.93–1.52)	0.16	0.75	0.0059
	Hypersomnia	753	1.04(0.71–1.51)	0.85	0.91	0.00016
ASD	Insomnia	750	0.91(0.77–1.07)	0.25	0.75	0.0026
	Restless sleep	526	0.90(0.75–1.07)	0.24	0.75	0.0033
	Sleep quality	527	0.80(0.64–0.99)	0.044	0.66	0.012
	Nightmares	527	0.96(0.75–1.24)	0.78	0.90	0.00025
	Hypersomnia	755	1.18(0.84–1.66)	0.33	0.83	0.0035
MDD	Insomnia	750	1.05(0.88–1.24)	0.60	0.90	0.00057
	Restless sleep	526	1.05(0.88–1.26)	0.57	0.90	0.00081
	Sleep quality	527	0.93(0.75–1.15)	0.48	0.90	0.0013
	Nightmares	527	1.01(0.81–1.26)	0.94	0.94	0.000014
	Hypersomnia	755	1.24(0.87–1.77)	0.23	0.75	0.0059

*FDR* false discovery rate, *R*^2^ variance explained by PGS, *ASD* Autism Spectrum Disorder, *MDD* Major Depressive Disorder

Sensitivity analyses using only CAPA information from parents, including additional covariates (sex and ADHD medication) and including only children not taking any sleep medication showed a similar pattern of results (Tables S3–S5).

## Discussion

In this study, we used PGS to investigate associations between sleep disturbances and ADHD using a clinical sample of children diagnosed with ADHD and their parents. First, we found that polygenic liability for long sleep duration was over-transmitted from parents to children with ADHD and this finding was supported in a combined analysis using an independent replication sample. Second, we did not find evidence that polygenic liability for sleep disturbances was associated with an increased risk of insomnia, poor sleep quality or hypersomnia in children with ADHD. Finally, we did not find evidence that greater genetic risk for ADHD or other frequently comorbid disorders were associated with increased risk of sleep disturbances in children with ADHD.

We found weak evidence that children with ADHD over-inherit polygenic liability for longer sleep duration. Both long and short sleep duration have previously been phenotypically associated with ADHD [34]. Prolonged sleep duration is a symptom of hypersomnia, which is common in people with ADHD [35]. Other evidence pointing to a shared pathophysiology comes from research on narcolepsy; both narcolepsy and ADHD can be treated using psychostimulants such as methylphenidate [36] and the prevalence of ADHD in children with narcolepsy is greater than in the general population [37]. Moreover, narcolepsy PGS have been associated with ADHD traits in the general population [10]. One theory is that inattentive and hyperactive-impulsive symptoms are a coping mechanism for deficits in alertness, but research supporting this is mixed [38]. Although our finding of over-transmission of sleep duration polygenic liability was not significant in an independent, larger ADHD sample, the direction of the effect was the same and the result was significant in analysis combining both samples. The effect size was stronger in the primary sample compared to the replication sample or combined analysis. This may be due to differences in the two samples; for example, fewer children in the IMAGE replication sample had sleep problems compared to the primary sample.

We did not find evidence in the primary or replication samples that children with ADHD over- or under-inherit polygenic liability for other sleep traits, specifically insomnia or chronotype. This did not support our hypothesis that sleep disturbances in ADHD arise due to shared genetic influences and contrasts with the previously reported genetic correlation between ADHD diagnosis and insomnia [8]. This could be due to differences in methodology, given we compared polygenic liability for insomnia in children to their parents’ average liability, rather than to screened controls. Our results also contrast with previous PGS studies in children in the general population, which found genetic links between ADHD and sleep phenotypes [9–11]. One possibility is that previous results were influenced by the fact that ADHD and sleep problems co-occur in the general population, which was not accounted for. Our results indicate that when potential confounders (e.g. ancestry, socioeconomic background) are taken into account using a trio design, there is limited evidence of shared genetic effects between childhood diagnosed ADHD and sleep phenotypes measured in adults.

Our results also indicated that polygenic liability for sleep disturbances was not associated with an increased risk of insomnia, poor sleep quality, or hypersomnia in children with ADHD. One explanation is that existing GWAS of sleep problems in adults do not index sleep problems in childhood, which extends to sleep problems in the context of clinically-diagnosed ADHD. The GWAS summary statistics we used are the largest available GWAS of sleep phenotypes but were derived from the UK Biobank, which consists of adults aged 40–69 years [39]. There is emerging evidence that the heritability of sleep traits changes across the lifespan [40, 41] and it is possible that the genetic architecture of sleep phenotypes is different in adults compared to children, regardless of ADHD diagnosis. However, currently available sleep GWAS in children are based on much smaller samples (e.g. [42]). Another possibility is that participants in our primary sample ranged in age from 5 to 18 years, whereas a recent study found that the association between ADHD and sleep disturbances peaked between 8 and 10 years of age [43]. It is, therefore, possible that genetic associations would also be greatest in this age range. In addition, we observed low rates of some sleep disorders in our sample (e.g. only 31 children in our sample had hypersomnia). This could be due to the interview method used to assess sleep phenotypes, or because a high proportion of our sample was taking medication for ADHD or sleep. Rates of hypersomnia might increase as children with ADHD become adults; it would therefore be pertinent to investigate associations between polygenic liability for adult sleep traits and sleep disturbances in adult samples with ADHD.

We did not find support for our hypothesis that greater polygenic liability for ADHD increased the risk of sleep disturbances in children with ADHD. This suggests that unlike other phenotypes (e.g. conduct disorder) [16], sleep disturbances in children with ADHD do not index a greater burden of risk for ADHD. It is possible that sleep disturbances in ADHD arise due to other factors, which should be investigated further. For example, children with ADHD are more likely to live in families of lower socioeconomic status [44], which is a risk factor for poor sleep and daytime sleepiness in children [13]. Symptoms of ADHD may make it more difficult for children and their parents to adopt healthy sleep practices, such as regular bedtimes. Randomised controlled trials of sleep interventions that improve sleep practices have been shown to be effective in improving sleep and ADHD symptoms in children with ADHD [6, 7]. Medications to treat ADHD may also improve or disrupt sleep [45]; however, we observed the same pattern of results in sensitivity analyses adjusting for ADHD medication and excluding children taking medication for sleep.

Other explanations for the link between ADHD and sleep should be considered. First, in our study we examined common genetic variants for sleep traits, but it is possible that sleep disturbances comorbid with ADHD also arise due to rare or de novo genetic variants for sleep traits or ADHD. Second is the role of gene-environment interplay; sleep disturbances in children with ADHD could arise in those who have a genetic predisposition to sleep problems and are exposed to particular environmental risk factors. Third is the role of power as the PGS were based on examining a small fraction of the genome (tens of thousands of SNPs) and our sample is much smaller than those typically used in GWAS and genome-wide genetic correlation analyses. Finally, disorders such as ASD, oppositional defiant disorder, anxiety, and depression are highly comorbid with ADHD [46, 47]. These disorders are also associated with sleep problems [48, 49] and may drive associations between ADHD and sleep disturbances. However, in this study, we did not find evidence that sleep problems in children with ADHD were driven by polygenic liability for ASD, MDD or anxiety. Future research needs to explore the impact of these comorbidities on sleep disturbances in children with ADHD.

### Limitations

Our study has several limitations, in addition to those outlined above. We measured sleep phenotypes using an interview, and a combination of parent and child-ratings. Some studies have shown discrepancies between parent and child reports of sleep, and their association with genetic factors; Breitenstein et al. [50] found that the heritability of sleep phenotypes in children differed depending on measurement method (self-report, parent-report or actigraphy). Future research should explore associations between ADHD and sleep using multiple measures of sleep phenotypes.

Our study also lacked phenotypic measures of chronotype, sleep duration, and other sleep disorders previously associated with ADHD [1], therefore we could not examine associations between ADHD/sleep PGS and these phenotypes. We were unable to test in our sample whether children with ADHD (regardless of sleep difficulties) had a higher burden of polygenic liability for sleep phenotypes, as we did not have a comparison sample of children without ADHD. Sleep disturbances were assessed when 30% of the sample were taking medication for ADHD and 15% were taking sleep medication, which may have influenced our results, although our sensitivity analyses indicated this is unlikely to fully explain our results. Our study may also have been underpowered due to only having 328 trios in the primary sample and 844 trios in the replication sample. Detailed sleep phenotypes were not available in the replication sample.

### Conclusions

Sleep problems are common in children with ADHD and impact on illness course, quality of life and family relationships [2–4]. Understanding why these sleep problems occur is important for understanding the aetiology of ADHD and identifying individuals who may be at higher risk of developing these problems. Using two large samples of children with ADHD, we find limited evidence for over-transmission of polygenic liability for sleep phenotypes, with weak evidence that polygenic liability for long sleep duration may be over-transmitted from parents to children. Our results also suggest comorbid sleep problems are not driven by genetic factors linked to sleep phenotypes measured in adults, or driven by polygenic liability for ADHD or other commonly comorbid neurodevelopmental and psychiatric disorders. However, our analyses utilised data from adult sleep GWAS and need to be extended using data from sleep GWAS in children, once sufficiently large samples are available. Further exploration of the role of sleep comorbidities in individuals with ADHD, and examination of this relationship using a wider range of sleep phenotypes is also needed. Our findings also highlight the need for additional exploration of biological and environmental factors that may give rise to sleep disturbances in children with ADHD.

## Supplementary Information

Below is the link to the electronic supplementary material.

Supplementary file1 (DOCX 299 KB)

## Data Availability

The code that supports the findings of this study is available on request from the corresponding author (KL).
