# Supplementary material for: Sleep disturbances in ADHD: investigating the contribution of polygenic liability for ADHD and sleep-related phenotypes
Source: Eur Child Adolesc Psychiatry. 2022 Jan 7;32(7):1253–61. doi: 10.1007/s00787-021-01931-2 (PMC10276090; doi:10.1007/s00787-021-01931-2)
Supplement: Supplementary file 1 — Supplementary file1 (DOCX 299 KB) [file 787_2021_1931_MOESM1_ESM.docx]

# **Supplemental Materials**

#

**Table S1: Results of the pTDT analysis in the primary, replication, and combined samples.**

| **Polygenic score** | **Primary ADHD sample** | | | | **Replication ADHD sample** | | | **Combined ADHD sample** | | |
| --- | --- | --- | --- | --- | --- | --- | --- | --- | --- | --- |
|  | **mean** | **SE** | **p** | **p_FDR_** | **mean** | **SE** | **p** | **mean** | **SE** | **p** |
| **Insomnia** | 0.080 | 0.057 | 0.16 | 0.27 | 0.013 | 0.032 | 0.68 | 0.032 | 0.028 | 0.25 |
| **Sleep duration** | 0.13 | 0.053 | 0.013 | **0.032** | 0.032 | 0.033 | 0.32 | 0.060 | 0.028 | **0.030** |
| **Chronotype** | 0.051 | 0.057 | 0.37 | 0.65 | 0.005 | 0.031 | 0.87 | 0.018 | 0.028 | 0.51 |
| **Sleep duration (long)** | 0.14 | 0.051 | 0.0076 | **0.032** | 0.060 | 0.034 | 0.077 | 0.081 | 0.028 | **0.0039** |
| **Sleep duration (short)** | -0.025 | 0.055 | 0.64 | 0.47 | 0.002 | 0.031 | 0.95 | -0.006 | 0.027 | 0.83 |

pTDT: polygenic transmission disequilibrium test; FDR: false discovery rate correction for multiple testing. Results in bold are significant after multiple testing correction or in the combined sample analysis.

#

# **Table S2: IMAGE sample description**

| **Binary phenotypes** | **N (%)** |
| --- | --- |
| Sex (female) | 111 (13.2) |
| Sleep problems | 169 (20.0) |
| ADHD medication use | 650 (77.0) |
| **Continuous phenotypes** | **Mean (SE)** |
| Child’s age at assessment | 10.9 (0.1) |
| Conners’ T Score (parent) | 77.1 (0.3) |
| Conners’ T Score (teacher) | 69.4 (0.4) |

Sleep problems assessed using the Parental Account of Childhood Symptoms (PACS) questionnaire, in which parents were asked “What about sleeping? Does X sleep well? Does he/she have any difficulty going to sleep, nightmares, sleepwalking?”. Here we have dichotomised responses as follows: 0=”no sleep difficulties”/”Little distress, no interference with daytime activities”, 1=”Moderate distress or moderate interference with daytime activities”/”marked distress or marked interference with daytime activities”. Conners’ T scores are prorated scores (raw scores adjusted for non-answered items) which have been normalised for age and sex and rescaled so that a population sample would be expected to have a distribution with a mean of 50 and standard deviation of 10. Further details on the coding algorithm used are available on the IMAGE website ([www.ncbi.nlm.nih.gov/projects/gap/cgi-bin/study.cgi?study_id=phs000016.v2.p2](https://www.ncbi.nlm.nih.gov/projects/gap/cgi-bin/study.cgi?study_id=phs000016.v2.p2))

**Table S3: Sensitivity analysis: Association of sleep and ADHD polygenic scores with sleep disturbances (parent-only ratings) in children with ADHD.**

| **Polygenic score** | **Outcome** | **N** | **OR (95% CI)** | **p** | **R^2^** |
| --- | --- | --- | --- | --- | --- |
| Insomnia | Insomnia | 750 | 1.11 (0.93-1.33) | 0.25 | 0.0029 |
|  | Poor sleep quality | 527 | 1.28 (1.00-1.64) | 0.052 | 0.012 |
| Sleep duration | Insomnia | 750 | 0.94 (0.79-1.12) | 0.51 | 0.00089 |
|  | Poor sleep quality | 527 | 1.07 (0.84-1.36) | 0.59 | 0.00088 |
|  | Hypersomnia | 754 | 1.14 (0.72-1.80) | 0.58 | 0.002 |
| Sleep duration (long) | Insomnia | 750 | 1.26 (1.07-1.49) | 0.0061 | 0.014 |
|  | Poor sleep quality | 527 | 1.21 (0.94-1.56) | 0.14 | 0.0077 |
|  | Hypersomnia | 754 | 0.96 (0.65-1.42) | 0.84 | 0.0002 |
| Sleep duration (short) | Insomnia | 750 | 1.12 (0.94-1.34) | 0.20 | 0.0034 |
|  | Poor sleep quality | 527 | 1.13 (0.90-1.41) | 0.29 | 0.003 |
|  | Hypersomnia | 754 | 0.91 (0.61-1.34) | 0.63 | 0.0011 |
| ADHD | Insomnia | 750 | 1.07 (0.90-1.26) | 0.44 | 0.0011 |
|  | Restless sleep | 524 | 0.97 (0.82-1.15) | 0.70 | 0.00037 |
|  | Poor sleep quality | 527 | 0.88 (0.70-1.12) | 0.31 | 0.0034 |
|  | Hypersomnia | 754 | 1.22 (0.77-1.93) | 0.41 | 0.0044 |

Covariates included in the models: top 5 principal components related to ancestry, genotyping batch and child’s age at assessment.

**Table S4: Sensitivity analysis: Association of sleep and ADHD polygenic scores with sleep disturbances in children with ADHD (including sex and ADHD medication as additional covariates).**

| **Polygenic score** | **Outcome** | **N** | **OR (95% CI)** | **p** | **R^2^** |
| --- | --- | --- | --- | --- | --- |
| Insomnia | Insomnia | 750 | 1.03 (0.86-1.24) | 0.71 | 0.00031 |
|  | Poor sleep quality | 527 | 1.16 (0.92-1.46) | 0.21 | 0.0047 |
| Sleep duration | Insomnia | 750 | 0.92 (0.78-1.10) | 0.37 | 0.0017 |
|  | Poor sleep quality | 527 | 1.06 (0.84-1.33) | 0.62 | 0.00072 |
|  | Hypersomnia | 755 | 1.02 (0.70-1.48) | 0.92 | 0.000053 |
| Sleep duration (long) | Insomnia | 750 | 1.14 (0.96-1.35) | 0.13 | 0.0045 |
|  | Poor sleep quality | 527 | 1.08 (0.85-1.36) | 0.54 | 0.0013 |
|  | Hypersomnia | 755 | 0.88 (0.63-1.23) | 0.45 | 0.0022 |
| Sleep duration (short) | Insomnia | 750 | 1.09 (0.92-1.29) | 0.34 | 0.0019 |
|  | Poor sleep quality | 527 | 1.10 (0.88-1.37) | 0.42 | 0.0019 |
|  | Hypersomnia | 755 | 0.89 (0.63-1.24) | 0.48 | 0.0019 |
| ADHD | Insomnia | 750 | 1.03 (0.87-1.22) | 0.75 | 0.0002 |
|  | Restless sleep | 526 | 0.93 (0.77-1.11) | 0.42 | 0.0018 |
|  | Poor sleep quality | 527 | 0.93 (0.75-1.15) | 0.49 | 0.0014 |
|  | Hypersomnia | 755 | 1.02 (0.69-1.52) | 0.91 | 0.000062 |

Covariates included in the models: top 5 principal components related to ancestry, genotyping batch, sex, ADHD medication use and child’s age at assessment.

#

**Table S5: Sensitivity analysis: Association of sleep and ADHD polygenic scores with sleep disturbances in children with ADHD (including only children not taking any sleep medication).**

| **Polygenic score** | **Outcome** | **N** | **OR (95% CI)** | **p** | **R^2^** |
| --- | --- | --- | --- | --- | --- |
| Insomnia | Insomnia | 438 | 0.92 (0.73-1.17) | 0.50 | 0.0017 |
|  | Poor sleep quality | 435 | 1.08 (0.85-1.37) | 0.54 | 0.0012 |
| Sleep duration | Insomnia | 438 | 0.99 (0.78-1.25) | 0.93 | 0.000031 |
|  | Poor sleep quality | 435 | 1.07 (0.84-1.38) | 0.57 | 0.0011 |
|  | Hypersomnia | 439 | 0.77 (0.47-1.26) | 0.31 | 0.0081 |
| Sleep duration (long) | Insomnia | 438 | 1.12 (0.89-1.41) | 0.32 | 0.0034 |
|  | Poor sleep quality | 435 | 1.05 (0.80-1.36) | 0.74 | 0.00041 |
|  | Hypersomnia | 439 | 0.76 (0.50-1.16) | 0.20 | 0.01 |
| Sleep duration (short) | Insomnia | 438 | 1.06 (0.84-1.33) | 0.64 | 0.00077 |
|  | Poor sleep quality | 435 | 1.07 (0.85-1.35) | 0.58 | 0.00093 |
|  | Hypersomnia | 439 | 0.91 (0.64-1.30) | 0.61 | 0.0011 |
| ADHD | Insomnia | 438 | 1.03 (0.83-1.28) | 0.79 | 0.00024 |
|  | Restless sleep | 434 | 0.91 (0.75-1.10) | 0.31 | 0.0032 |
|  | Poor sleep quality | 435 | 0.89 (0.70-1.13) | 0.32 | 0.0032 |
|  | Hypersomnia | 439 | 0.91 (0.60-1.38) | 0.66 | 0.0011 |

Covariates included in the models: top 5 principal components related to ancestry, genotyping batch and child’s age at assessment.

#

# **Supplemental Text**

## *Primary ADHD Sample (SAGE)*

Children were genotyped for a GWAS of ADHD [(Stergiakouli et al. 2012)](https://paperpile.com/c/XVWCqc/s0acE) using the Illumina Human660W-Quad BeadChip. Additional children and parents were genotyped using a customised version of the PsychChip. Batch-specific QC included aligning SNPs to the Haplotype Reference Consortium data using GenomeHarmoniser, removing SNPs with a minor allele frequency (MAF) <1%, genotyping rate<0.95, or HWE p<10^-6^, and removing individuals if they had missingness >5%, sex discrepancy, or if they were duplicate samples. Samples using the same genotyping platform were merged, QC parameters reapplied, and the data were imputed using the Michigan Imputation Server (using Eagle v2.4 for phasing, Minimac4 for imputation, and the HRC V1.1 imputation reference panel). Imputed dosage data were converted to best guess genotype data using PLINK version 1.9 [(Purcell et al. 2007)](https://paperpile.com/c/XVWCqc/ZRGL) and post-imputation QC filters were applied as follows: genotype probability >0.9 per individual, missingness <0.03, MAF>0.01, HWE>10^-4^, and INFO/r2>0.8. Identity-by-descent (IBD) and Mendel analyses in PLINK were used to confirm family relationships and individuals who were not related as expected were excluded. Variants with >5 Mendel errors were excluded, and all remaining Mendel errors were set to missing. We performed a principal components analysis (PCA), on LD-pruned common (MAF>5%) SNPs using PCAiR [(Conomos, Miller, and Thornton 2015)](https://paperpile.com/c/XVWCqc/NnZg3), a package that robustly estimates population structure while taking into account kinship information in the sample. Samples of non-European ancestries were excluded. SNPs indicating batch effects (p<0.01 in a GWAS of genotyping batch using unrelated samples) were excluded. PCAiR of the final dataset was used to extract PCs to use as covariates. Complete parent-offspring trios consisted of families with one proband and both biological parents, who were all genotyped using the PsychChip and passed QC. A total of 3,335,041 SNPs and 857 children with ADHD, including 328 complete parent-offspring trios, passed all of the above filters and QC.

*PGS calculation*

For each of these GWAS, common variants (MAF>5%) that overlapped with our sample were LD-clumped in PLINK (--clump-kb 500 --clump-r2 0.2) to obtain an independent set of SNPs, while retaining the most significant SNP in each LD block. PGS were calculated for each individual by summing the number of alleles (weighted by the log of the odds ratio) across the set of SNPs in PLINK (using the command --score) and standardised using z-score transformations. Scores were calculated using 7 p-value thresholds to select SNPs (p_T_<1, p_T_<0.5, p_T_<0.1, p_T_<0.05, p_T_<0.01, p_T_<0.001, p_T_<0.00001). We then used the PGS-PCA approach [[29]](https://paperpile.com/c/zF7iCb/nXdV), in which a principal component analysis (PCA) was conducted on scores from the 7 p-value thresholds and the first principal component was extracted and used for analysis to test associations between the PGS and the outcomes of interest. Two children who were outliers for anxiety PGS (<-4SD) were excluded from the anxiety PGS analyses.

## *Replication ADHD Sample*

The presence or absence of each DSM-IV ADHD symptom was determined from responses to PACS questions using an algorithm [(Taylor et al. 1986)](https://paperpile.com/c/XVWCqc/Dfpf9). This information is combined with items scored 2 or 3 from the teacher-rated Conners’ ADHD scale [(Conners et al. 1998)](https://paperpile.com/c/XVWCqc/0UEWQ) in order to derive the total number of DSM-IV ADHD symptoms. Where possible, parents were asked to rate the child’s behaviour when they were not medicated.

The IMAGE data were accessed via dbGAP under approved Project #26394. After basic QC exclusions (SNP missingness >0.02, individual missingness >0.02, HWE threshold p<1-10, heterozygosity >4SD), data were converted to genome build hg19, and imputed using the Haplotype Reference Consortium (HRC) reference panel, using the Michigan imputation server. Best guess genotypes were defined using PLINK version 1.9, using the following QC filters: imputation info score >0.8, and MAF>0.05. Family relationships were confirmed using IBD and Mendel analyses in Plink and Mendel errors were set to missing. Only complete parent-offspring trios were kept for analyses. The sample is of broadly-defined European ancestry [(Neale et al. 2008)](https://paperpile.com/c/XVWCqc/HTgha) and no restrictions were made based on ancestry.

# **References**

[Conners, C. K., G. Sitarenios, J. D. Parker, and J. N. Epstein. 1998. “The Revised Conners’ Parent Rating Scale (CPRS-R): Factor Structure, Reliability, and Criterion Validity.” *Journal of Abnormal Child Psychology* 26 (4): 257–68.](http://paperpile.com/b/XVWCqc/0UEWQ)

[Conomos, Matthew P., Michael B. Miller, and Timothy A. Thornton. 2015. “Robust Inference of Population Structure for Ancestry Prediction and Correction of Stratification in the Presence of Relatedness.” *Genetic Epidemiology* 39 (4): 276–93.](http://paperpile.com/b/XVWCqc/NnZg3)

[Neale, Benjamin M., Jessica Lasky-Su, Richard Anney, Barbara Franke, Kaixin Zhou, Julian B. Maller, Alejandro Arias Vasquez, et al. 2008. “Genome-Wide Association Scan of Attention Deficit Hyperactivity Disorder.” *American Journal of Medical Genetics. Part B, Neuropsychiatric Genetics: The Official Publication of the International Society of Psychiatric Genetics* 147B (8): 1337–44.](http://paperpile.com/b/XVWCqc/HTgha)

[Purcell, Shaun, Benjamin Neale, Kathe Todd-Brown, Lori Thomas, Manuel A. R. Ferreira, David Bender, Julian Maller, et al. 2007. “PLINK: A Tool Set for Whole-Genome Association and Population-Based Linkage Analyses.” *American Journal of Human Genetics* 81 (3): 559–75.](http://paperpile.com/b/XVWCqc/ZRGL)

[Stergiakouli, Evangelia, Marian Hamshere, Peter Holmans, Kate Langley, Irina Zaharieva, deCODE Genetics, Psychiatric GWAS Consortium, et al. 2012. “Investigating the Contribution of Common Genetic Variants to the Risk and Pathogenesis of ADHD.” *The American Journal of Psychiatry* 169 (2): 186–94.](http://paperpile.com/b/XVWCqc/s0acE)

[Taylor, E., R. Schachar, G. Thorley, and M. Wieselberg. 1986. “Conduct Disorder and Hyperactivity: I. Separation of Hyperactivity and Antisocial Conduct in British Child Psychiatric Patients.” *The British Journal of Psychiatry: The Journal of Mental Science* 149 (December): 760–67.](http://paperpile.com/b/XVWCqc/Dfpf9)
